# Obstetrics and Diseases of Women

**Published:** 1893-04

**Authors:** W. Keiller

**Affiliations:** F. R. C. S., Ed.; Texas Medical College, Galveston, Texas


### OBSTETRICS AND DISEASES OF WOMEN.

W. KEILLER, F. R. C. S., ED.
Texas Medical College, Galveston, Texas.

### VESICO-CERVICAL FISTULA.

Jacob Rosenthal reports from Prof. Leopold's clinic, at Dresden, an interesting case of the cure of vesico-cervical urinary fistula (complicated by a sinus leading to a carious focus in the pubic symphysis) by the supra pubic method.

The patient being in Trendelenburg's position, a transverse incision four inches long, parallel with the symphysis, is made through the recti; the bladder loosened from behind the pubes, a transverse incision in the anterior bladder wall; the bladder flaps stitched temporarily to the abdominal walls, and the fistula treated through this means of approach. A catheter is retained in the bladder, and supra-pubic drainage is practiced. The method is valuable as a substitute for the older method of closing the cervix uteri and converting uterus and bladder into one cavity, menstruation then taking place per urethram.—*Am. Jour. Obstet. and Dis. of Women*, March, 93.

---

CONGENITAL MALFORMATION OF FEMALE GENITAL ORGAN.

Dr. Munde reports a number of interesting cases of the above, including:

1. A case of rudimentary uterus, absent ovaries, well developed vulva, but entire absence of vagina. He made a vagina, and stitched the uterus to its upper end.

2. Two cases who had not menstruated up to eighteen and twenty years. Each had a perfect vagina, but in one the uterus was rudimentary, in the other absent. Treatment, of course, was useless.

3. Case of double uterus and vagina. Menstruation from left half regular; imperforate hymen on right side, and retained menstrual fluid.

4. Case of perfect double uterus and vagina, accompanied by dysmenorrhœa which resisted all ordinary treatment. Oophorectomy had been advised. Finding the appendages normal, Munde advised the removal of the septum between the two halves of the vagina and uterus, up to the fundus. This was followed by perfect restoration to health.

The same treatment was followed in case 3, with excellent results.

5. Case of double uterus. Patient had one child, which had from the subsequent history been evidently borne by the more developed half. Pregnancy in the more rudimentary horn was first taken for a tubal pregnancy, and cœliotomy performed. The appendage being found normal Dr. Munde then thought it an intra-mural gestation, but more careful examination showed that it was double uterus. Wound stitched up. Patient aborted; recovery.—*Amer. Jour. Obstet and Dis. Wom. March, 1893.*

---

RECENT LITERATURE OF PUERPERAL ECLAMPSIA.

In a review of the above in the *American Journal of Obstetrics and Diseases of Women*, (March, 1893,) the balance of evidence is against puerperal eclampsia being due to any specific microorganism. The usual cause is toxines retained in the system from deficient kidney action—in most cases albuminuria is present.

In 93 75 % of cases the convulsions cease on delivery, and speedy delivery is therefore the best treatment. Duhrssen urges the induction of labor by rapid dilatation, aided if necessary, by deep cervical incisions; and condemn the protracted use of chloroform, chloral and morphine. Meckel also urges early delivery, reporting cases favoring this practice.

---

THE INDUCTION OF PREMATURE LABOR BY INTRA-UTERINE INJECTIONS OF GLYCERINE.

This method of inducing premature labor has attracted considerable attention lately. Pelzer describes it as follows:

Patient in Sim's position. A syringe with long nozzle (or gum-elastic catheter) is introduced between the membranes and the uterus, and about 150 grammes (five ounces) of pure glycerine is slowly injected, care being taken to empty the syringe or catheter of air. The glycerine causes mechanical separation and acts as an irritant, and its hygroscopic properties cause transudation of the liquor amni. It is safe and prompt.—*Archiv. for Gynakologu Bd. XLIL. Heft. 2.*

---

Thos. Addis Emmet in an article on Female Plastic Surgery, laments the tendency to do multiple gynæcological operations at the same sitting; especially to repair a lacerated cervix and the perinæal floor at the same operation.

Regarding repair of the lacerated cervix, he thinks that more harm than good has resulted from it in the hands of the profession at large.

To do it properly is more difficult than an average ovariotomy Yet in well selected cases the operation, if properly performed, will cause rapid involution and, relieve many reflex disturbances

Of operations for lacerated perinæum, he condemns all operations which merely place a bridge of skin on part of the descending recto-cecle. The pathology of the condition producing a recto-cecle is a separation and retraction of the vaginal and rectal attachments of pelvic fascia and levatores ani; and this is to be restored by narrowing the posterior vaginal wall, and bringing it up towards the anterior—thus approximating the separated insertions of the pelvic fascia and levatores ani.

Vesico vaginal fistula he finds mainly caused by delay in dellvery, and especially by the use of ergot. He has never seen it caused by instrumental delivery. It has become uncommon since ergot has been given up in the first and second stages, and instruments have been the rule before impaction of the head. Dr. Emmet has succeeded in restoring the base of the bladder and entire floor of the urethra after extensive sloughing from delayed delivery. He attributes failure to cure vesico-vaginal fistula to three causes. 1st. An effort to unite parts requiring too much tension to approximate them. 2d. To improper and too tight twisting of the wire sutures. 3d. To the use of a catheter not properly suited to the individual case. He advocates strongly silver wire and Sims' self retaining catheter.

Silver wire is especially adapted to plastic operations and abdominal sections, because no form of suture will keep such broad surfaces in apposition as silver wire properly applied—it is at once a splint and a suture.

The avoidance of all cicatricial material in the vagina or genital tract wherever there is erectile tissue, is of the utmost importance, as cicatrices anywhere in this situation may cause reflex disturbances. The first step in the closure of vesico-vaginal fistula is to free all bands causing tension on the parts. This must be done by antero posterior section of transverse bands, and immediate closure of the wound, uniting the anterior and posterior angles of the resulting wound so as to produce a transverse line of sutures, and healing by first intention must be secured.

He condemns flap-splitting operations for the restoration of recto-vaginal septums, emphasises the necessity for union of the separated levatores ani and sphincter, and reiterates the efficiency of his own operation described in 1873. (See gynæcological text books.)—*New York Journal of Gynæcology and Obstetrics. February, 1893.*

